# Cloning of the Human C5a Anaphylatoxin Receptor, and More

**DOI:** 10.3389/fimmu.2015.00445

**Published:** 2015-09-01

**Authors:** Norma P. Gerard, Craig Gerard

**Affiliations:** ^1^Ina Sue Perlmutter Laboratory, Division of Respiratory Diseases, Department of Medicine, Children’s Hospital, Harvard Medical School, Boston, MA, USA; ^2^Department of Medicine, Beth Israel Deaconess Medical Center, Boston, MA, USA

**Keywords:** C5aR/C5R1, 7-TMS receptors, complement anaphylatoxin, C5a, G protein coupled receptor

The initial observation in 1973 that the complement C5 activation product, C5a, has the ability to stimulate human neutrophils led to the concept of a specific C5a receptor (1). In the mid-1986, a groundbreaking paper published in Nature announced the cloning of the beta-adrenergic receptor, and for the first time established seven transmembrane (7TM) G protein-coupled receptors as members of the rhodopsin superfamily (2). Almost simultaneously, Feltner and colleagues demonstrated that the FMLP, C5a, and LTB4 activities on rabbit neutrophils could be inhibited by pertussis toxin, indicating coupling to GTP binding proteins (3). In 1987, Masu and colleagues used an oocyte expression cloning system to isolate a cDNA encoding the neuropeptide substance K receptor; and later, the same group identified the related substance P receptor (4, 5).

In 1989, we realized that the pertussis sensitivity of the fMLP, C5a, and LTB4 receptors suggested that they would also be members of the rhodopsin superfamily. When we aligned the handful of structures for the then known 7TM receptors (adrenergic, serotonin, dopamine, FSH/LH, and substance P and K receptors), we recognized homologies in both the transmembrane segments and intracellular loops, which presumably facilitated interactions with G proteins. This observation actually presented us with an opportunity to attempt to clone receptors by homology to the superfamily as “orphan receptors.”

We constructed an antisense oligonucleotide with minimal degeneracy that encompassed a highly conserved NPXXY motif in the seventh transmembrane segment of the known rhodopsin family members. In order to enrich in C5a receptors, we took advantage of the fact that the receptors were induced by cyclic-AMP in U937 cells, and in retinoic acid differentiated human HL60 cells. By summer of 1990, we had isolated ~20 cDNAs using this approach from the cAMP induced U937 cell library. About half of these clones were an identical cDNA that we named NPIIY-18. Using this as a probe, we demonstrated that NPIIY-18 recognized a ~2.2 kb mRNA only in cAMP differentiated U937 cells. Northern blot analyses showed that NPIIY-18 was present only in cells known to express the C5a receptor. As NPIIY-18 was not a full-length cDNA, we then probed the retinoic acid differentiated HL60 cell library. We isolated a full-length DNA from this library that encoded a 7TM receptor with 25% homology to the substance K receptor and 35% homology to the human fMLP receptor (FPR1), which was cloned by Francois Boulay in May 1990 (6). When expressed in COS cells, we showed that NPIIY-18 encoded a high-affinity receptor for human C5a (Figure 1) (7). This work was accepted for publication in Nature in December 1990. In the summary paragraph of this manuscript, we pointed out that our approach should be helpful to clone the receptors for the leukotrienes, platelet activating factor, interleukin-8, and adenosine receptors as these are all present on cAMP differentiated U937 cells. Almost simultaneously, Francois Boulay confirmed our identification of the human C5aR, which his group accomplished by expression cloning of differentiated HL-60 cells (8). His work was published in March 1991, some 4 months after ours.

**Figure 1:**
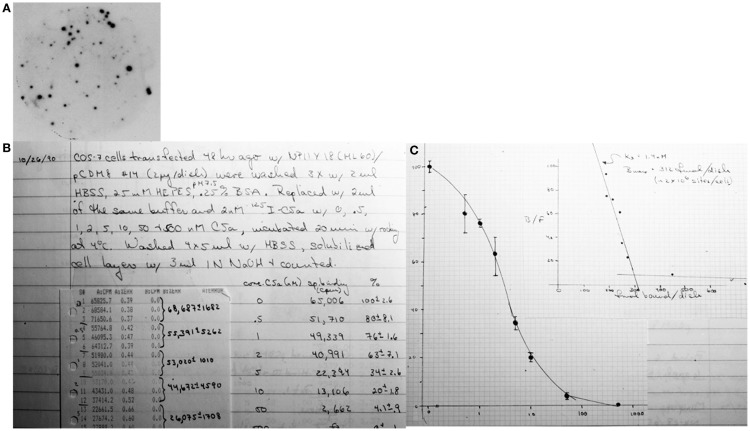
**Final steps in cloning and identifying the human C5a receptor**. **(A)** Tertiary screen of clone NPIIY-18 using ^32^P-labeled oligonucleotide probe. **(B)** NPIIY-18 was transfected into COS7 cells and tested for binding of ^125^I-C5a under competitive binding conditions. **(C)** The resulting binding isotherm and Scatchard analysis proving the clone’s identity.

In November 1990, within months of the identification of the human FPR1, Thomas et al. reported the cloning of the rabbit receptor for fMLP, F3R (9), which had almost no significant homology to the human receptor. This was troubling, because we knew that when the ligand was identical from species to species, the receptors were generally highly conserved. Thus, the adrenergic, dopamine, serotonin, and histamine receptors are >90% identical across species. Curiously, the cDNA was reported to bind the FPR1 radioligand and transduce calcium transients. Because the expression of the claimed rabbit F3R formyl peptide receptor was restricted to neutrophils, we wondered if, in fact, the Navarro lab had misidentified an interleukin-8 receptor. One of us (Craig Gerard) actually traveled to the Navarro lab to obtain the F3R cDNA to establish a collaboration and test its identity as a receptor for IL-8. At that time, there was no radioligand IL-8 commercially available. Henry Showell, of Pfizer Central Research, was able to provide us with a custom iodinated IL-8, which we demonstrated to bind F3R. Unfortunately, we did not have sufficient quantities of the reagent to perform comprehensive studies to publish our findings. We disclosed our result to Javier Navarro, but were left in silence. Unbeknownst to us, Dan Witt, at Repligen, had reportedly approached the Navarro lab with a similar idea. Thomas et al. went on to publish F3R as an IL8 receptor, without retracting the previous paper (10). During this time, Tom Schall and I met at a FASEB meeting with Phil Murphy, and suggested to him that he use F3R to clone a human homolog from HL60 cells and test it against IL8. The landmark Murphy and Tiffany paper resulted (11). Phil offered one of us (Craig Gerard) coauthorship for the helpful suggestion but because of intellectual property concerns at our institution, we requested an acknowledgment instead.

Over the next decade, the orphan receptor approach led to the identification of a wide variety of chemoattractant receptors, including most of the chemokine receptor system. The most notable events in the area of chemokines occurred when CXCR4 and CCR5 were identified as HIV coreceptors. It was known from the work of Ed Berger that CXCR4 was the obligate coreceptor with CD4 for laboratory-adapted strains of HIV (12). However, the wild type, the so-called macrophage tropic strain used an unknown coreceptor. In December 1995, it was reported in the New York Times that the Gallo laboratory had identified Mip1α, Mip1β, and RANTES as substances that inhibited HIV infections (13). Coincidentally, at the Fourth International Chemokine Symposium, held June 27–30, 1995, Izzy Charo described an orphan receptor identified as CCR5, linked to CCR2, which bound Mip1α, Mip1β, and RANTES (14). Thus, an international race began as five chemokine labs partnered with HIV labs to prove the hypothesis that CCR5 was the HIV coreceptor.

## Conflict of Interest Statement

The authors declare that the research was conducted in the absence of any commercial or financial relationships that could be construed as a potential conflict of interest.
